# Lamin A/C Cardiomyopathy with E203K Pathogenic Mutation

**DOI:** 10.7759/cureus.7761

**Published:** 2020-04-21

**Authors:** Fahad N Sheikh, Syed Adeel Hassan, Dilnaz Alam, Maryam Kundi, Moeez Hassan

**Affiliations:** 1 Pathology, Sahiwal Medical College, Sahiwal, PAK; 2 Neurology, Dow University of Health Sciences, Karachi, PAK; 3 Internal Medicine, Dow University of Health Sciences, Karachi, PAK; 4 Internal Medicine, Khyber Teaching Hospital, Peshawar, PAK; 5 Internal Medicine, Coliseum Medical Centers, Macon, USA; 6 Pediatrics, Beaumont Hospital, Royal Oak, USA

**Keywords:** cardiomyopathy, sudden death, atrioventricular block, lmna, pacemaker

## Abstract

Lamin A/C (LMNA) cardiomyopathy is an adult-onset, autosomal dominant, rapidly progressive cardiomyopathy which belongs to a spectrum of familial idiopathic cardiomyopathies. It is the most common type of familial dilated cardiomyopathy that is associated with conduction defects. A 76-year-old African American female with second-degree atrioventricular (AV) block presented for evaluation of persistent fatigue. Her family history was significant for sudden deaths of her son and brother at the age of 6 and 48 years, respectively, and AV block in her sister with a pacemaker implant at the age of 64 years. Physical examination was within normal limits. Electrocardiogram showed a Mobitz type II, second-degree AV block. Mild dilated cardiomyopathy was present on echocardiogram. Stress echocardiography had to be stopped due to premature ventricular contractions. Cardiac catheterization, coronary angiography, and cardiac MRI revealed no significant etiology for rhythm disturbance. Holter monitoring revealed intermittent bradycardia with a heart rate falling as low as 28 beats per minute, which led to the decision of dual-chamber pacemaker implantation. RhythmNext genetic testing (Ambry Genetics, Aliso Viejo, CA) was done due to the significant family history of sudden death; it revealed a heterozygous E203K pathologic mutation in the LMNA gene. Sudden death is the most common mode of death in LMNA cardiomyopathy; hence, the implantation of intracardiac cardioverter-defibrillator for primary prophylaxis was discussed with the patient. Clinicians should suspect LMNA cardiomyopathy in patients with rhythm disorders and family history of sudden death, which can help to identify individuals at risk and prevent sudden death by appropriate interventions.

## Introduction

Lamin A/C (LMNA) cardiomyopathy is a well-studied etiology of idiopathic dilated cardiomyopathy. The LMNA gene encodes for intermediate filament proteins nuclear lamin A and nuclear lamin C, which are components of the nuclear lamina [1]. LMNA gene-associated mutations can lead to well-defined diseases involving striated muscles, adipose tissue, peripheral nerves, or multiple systems with characteristics of accelerated aging. Cardiac involvement was first described as a part of Emery-Dreifuss muscular dystrophy (a triad of dilated cardiomyopathy, humero-temporal muscle weakness, and early tendon contractures) [2]. Later on, isolated defects in cardiac contractility and conduction due to missense mutations in LMNA gene were demonstrated [3].

LMNA-associated cardiac diseases have an adult-onset and aggressive course [4-6]. Isolated cardiac or musculoskeletal phenotypes have an extremely high penetrance among carriers. Almost 100% cardiac penetrance has been reported by the age of 60 years [6]. Some mutations affecting LMNA genes can also affect infants. Fetal cardiac involvement with LMNA mutations is frequent and fatal [7].

## Case presentation

The case we report here is of a 76-year-old African American female who presented with a complaint of gradually progressive, continuous fatigue which has limited her daily activities. There were no significant aggravating or alleviating factors for her tiredness. She denied any associated palpitations, dyspnea, orthopnea, paroxysmal nocturnal dyspnea, chest pain, cough, hemoptysis, pedal edema, blurry vision, abdominal pain, fever, chills, or night sweats, or any changes in weight, appetite, mood, or sleep patterns. Her past medical history was significant for syncope, hypertension, stage III chronic kidney disease, diverticulosis, hypothyroidism, and obesity. The patient also recalled an unprovoked syncopal episode 15 years ago that led to the diagnosis of second-degree atrioventricular heart block. Her medications at the time of initial presentation included aspirin (81 mg) and Synthyroid (25 mcg) daily. The patient was referred to the cardiology clinic for evaluation of Mobitz II, second-degree heart block. Her family history was significant for sudden cardiac death. Her son died at the age of six years, while her brother died suddenly at the age of 48 years. Her sister, 64 years old, has Mobitz type II, second-degree atrioventricular block that is treated with a pacemaker.

**Figure 1:**
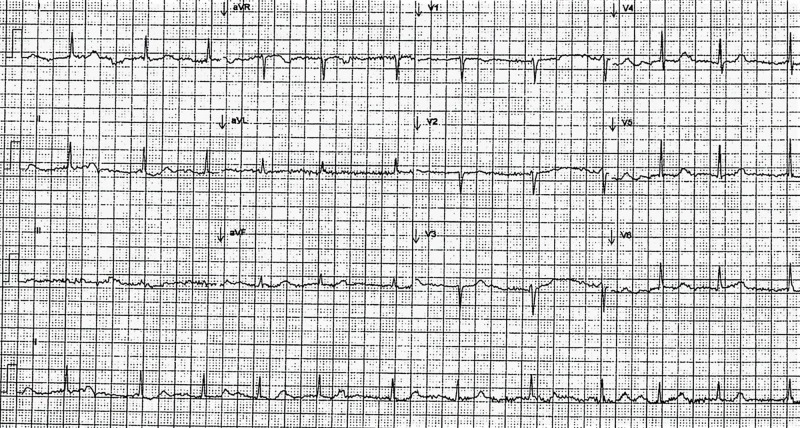
Electrocardiogram Lead II shows intermittent prolonging of R-R intervals, showing failure of atrioventricular conduction.

On general physical examination, her blood pressure was 164/80 mmHg while heart rate was 48 beats per minute and regular. The temperature, respiratory rate, oxygen saturation, and weight were within normal limits. Cardiac examination revealed unremarkable S1 and S2 heart sounds with no associated murmurs, rubs, or gallops. The point of maximal impulse was not displaced. She had clear breath sounds bilaterally. Abdominal, musculoskeletal, and neurologic examinations were unremarkable. Initial blood tests including complete blood count, basic metabolic panel, thyroid function tests, and Lyme serology were normal. Baseline electrocardiogram was normal except for a Mobitz type II, second-degree atrioventricular block (Figure 1). The patient was advised to monitor blood pressure periodically and keep a record of it. A follow-up appointment was scheduled for further investigations at the outpatient cardiology clinic.

Transthoracic echocardiogram estimated an ejection fraction of 60%-65% along with traces of mitral and tricuspid regurgitation and mild left ventricular dilatation with no evidence of diastolic dysfunction. On further follow-up, the patient underwent stress echocardiography due to the physician’s concern of significant family history of sudden cardiac death. She developed frequent premature ventricular contractions in couplets and triplets before reaching peak exercise intensity, which led to termination of the stress test. Because of the second-degree atrioventricular block and an abnormal stress test, left heart catheterization with selective coronary angiography was scheduled. The procedure was performed without any complications. Left ventricular systolic pressure was 160 mmHg, left ventricular end-diastolic pressure was 16 mmHg, and the aortic pressure was 159/59 mmHg. On angiography, the left ventricle revealed a large calcium deposit on the anterior wall but there was no evidence of coronary artery disease. To assess for infiltrative cardiomyopathy, the patient underwent cardiac MRI which revealed normal left ventricular global and segmental function with an ejection fraction of 65%. There was no evidence of myocardial scarring or infiltrative process. She was discharged with outpatient cardiac telemetry and Holter monitor.

The patient returned to the clinic two months later with a complaint of persistent fatigue described as tiredness and weakness. Her Holter monitor revealed intermittent complete heart block with the heart rate occasionally dropping to 28 beats per minute during day time. In order to avoid sudden death due to atrioventricular heart block, she was scheduled for a dual-chamber pacemaker implant. The procedure was successful, and the patient recovered without any adverse health effects.

In order to reach a conclusive diagnosis, the patient agreed upon genetic testing for inherited arrhythmias. Following genetic counseling, she underwent RhythmNext genetic testing (Ambry Genetics, Aliso Viejo, CA). The test revealed that the patient was heterozygous for the p.E203K pathogenic mutation in LMNA gene which is consistent with the diagnosis of LMNA-related cardiomyopathy. Due to the rarity of E203K mutation, the severity of the disease for this patient was unpredictable on the basis of the genetic test result. LMNA E203K mutation was subsequently found in her sister as well. The patient's daughter and another sister, both of whom are healthy, tested negative for this mutation.

At the latest follow-up, the patient's fatigue had subsided. She had not suffered from any adverse or unanticipated events due to her pacemaker implant. She was compliant with her medications which comprised of aspirin (81 mg once daily) and Synthyroid (25 mcg once daily). Intracardiac cardioverter-defibrillator implantation for primary prophylaxis of fatal arrhythmias was also discussed with the patient.

## Discussion

The case of LMNA E203K pathogenic mutation-associated cardiomyopathy is a rare type of isolated LMNA cardiomyopathy. LMNA mutations were detected in 7.5% cases of dilated cardiomyopathy with positive family histories and in 3.6%-11% cases of sporadic dilated cardiomyopathy [4,8]. There is a relatively higher prevalence (33%) of LMNA mutations in patients with familial dilated cardiomyopathy associated with conduction blocks [9]. It is unclear how many of these cardiomyopathy cases are due to the specific E203K mutation described in this case.

LMNA cardiomyopathy is an adult-onset autosomal dominant disease with 100% penetrance by the age of 60 years [2,6]. Data show that cardiac diseases due to LMNA mutations exhibit an aggressive course with high risk of heart failure and fatal arrhythmias [4-6]. Approximately 55% of patients with LMNA cardiomyopathy die or receive heart transplants by the age of 60 years, whereas only 11% of patients with isolated cardiomyopathy have similar outcomes [4].

Currently, no clinical criteria reliably distinguish LMNA cardiomyopathy from idiopathic dilated cardiomyopathy. There is a variable age of onset of cardiac signs and symptoms. A meta-analysis reported that arrhythmias and conduction defects precede contractile dysfunction and symptoms of heart failure [5]. Any level of the conduction system can be affected by LMNA mutations and manifest as atrioventricular block, bundle branch block, or sick sinus syndrome. Almost 46% of patients with LMNA mutations, regardless of phenotype, suffered from sudden death. Nearly 28% of LMNA gene mutation carriers received pacemakers, but no change in the rate of sudden death was noted [5].

Clear treatment guidelines are yet to be established for cases of LMNA cardiomyopathy with conduction system defects that has not progressed to heart failure. If an LMNA carrier receives a pacemaker due to conduction system defects, the placement of intracardiac cardioverter-defibrillator for primary prophylaxis of fatal arrhythmias is vital, despite the lack of gross dilated cardiomyopathy [10]. Treatment of clinically detectable dilated cardiomyopathy due to LMNA mutations follows the standard care of heart failure. There is not enough data to support the early institution of therapeutic agents like angiotensin-converting enzyme inhibitors, angiotensin receptor blockers, beta-blockers, and/or aldosterone antagonists in LMNA carriers.

## Conclusions

Isolated LMNA cardiomyopathy with E203K pathogenic mutation is extremely rare. There is no reliable clinical criteria to distinguish LMNA cardiomyopathy from idiopathic cardiomyopathy. Thorough investigation including genetic testing of idiopathic conduction defects, despite a lack of significant heart failure, is vital in diagnosing LMNA cardiomyopathy. Timely diagnosis will be helpful in identifying individuals at risk. It will also enable clinicians to discuss intracardiac cardioverter-defibrillator implantation to prevent sudden deaths.
